# Long Term Follow-Up of the Endovascular Trans-Vessel Wall Technique for Parenchymal Access in Rabbit with Full Clinical Integration

**DOI:** 10.1371/journal.pone.0023328

**Published:** 2011-08-16

**Authors:** Johan Lundberg, Stefan Jonsson, Staffan Holmin

**Affiliations:** 1 Department of Clinical Neuroscience, Karolinska Institutet and Department of Neuroradiology, Karolinska University Hospital, Stockholm, Sweden; 2 Department of Materials Science and Engineering, Royal Institute of Technology, Stockholm, Sweden; Stanford University Medical Center, United States of America

## Abstract

**Objective:**

Endovascular techniques are providing options to surgical/percutaneous cell transplantation methods. Some cells, e.g. insulin producing cells, are not suitable for intra-luminal transplantation and for such cells, other options must be found. We have constructed a “nanocatheter” with a penetrating tip for vessel perforation, thereby creating a working channel for parenchymal access by endovascular technique. To finish the procedure safely, the distal tip is detached to provide a securing plug in the vessel wall defect.

**Materials and Methods:**

We have performed interventions with full clinical integration in the superior mesenteric artery (SMA), the subclavian artery and the external carotid artery in rabbits. No hemorrhagic- or thromboembolic events occurred during the procedure. Stenosis formation and distal embolisation were analyzed by angiography and macroscopic inspection during autopsy at five, 30 and 80 days. All animals and implanted devices were also evaluated by micro-dissections and histochemical analysis.

**Results:**

In this study we show safety data on the trans-vessel wall technique by behavioral, angiographical and histological analysis. No stenosis formation was observed at any of the follow-up time points. No animals or organs have shown any signs of distress due to the intervention. Histological examination showed no signs of hemorrhage, excellent biocompatibility with no inflammation and a very limited fibrous capsule formation around the device, comparable to titanium implants. Further, no histological changes were detected in the endothelia of the vessels subject to intervention.

**Conclusions:**

The trans-vessel wall technique can be applied for e.g. cell transplantations, local substance administration and tissue sampling with low risk for complications during the procedure and low risk for hemorrhage, stenosis development or adverse tissue reactions with an 80 days follow-up time. The benefit should be greatest in organs that are difficult or risky to reach with surgical techniques, such as the pancreas, the CNS and the heart.

## Introduction

Cell based therapies are in clinical trials in *e.g.* Parkinsons disease [1], [2], ischemic stroke [3], [4] and spinal cord lesions [5]. Outside the CNS, other clinical trials involving *i.e.* muscle dystrophy [6], [7], ischemic heart disease [8], graft versus host disease [9], [10] and type I diabetes mellitus [11], [12] have yielded promising results.

Endovascular based specific intra-arterial approaches have previously been shown to achieve superior engraftment levels vis-à-vis intravenous methods [13], [14]. However, not all cells are even possible to engraft by intraluminal methods as illustrated by a specific intra-arterial approach to insulin producing cell transplantation to the pancreas [15]. For cell transplantation schemes aimed at organs that are difficult or hard to reach, such as the pancreas, the CNS, the heart and the lung, endovascular methods can provide a minimally invasive option. An example of such transplantations is the percutaneous approach to transplantation of insulin-producing cells via the portal vein. It has been speculated that it is dependent on transplanted cell acting as embolic material in the low pressure system that the portal vein constitutes [16]. Nevertheless, the portal vein approach to transplantation is today the most promising one due to the risks of existing open surgical options [17], [18]. Transplantation of insulin producing cells to the pancreas itself appears to come with favorable conditions such as the higher oxygen tension in the pancreatic tissue compared to the liver. Further, superior endocrine glucose regulation capacity can be reached with cells situated in the pancreas compared to the liver. It would therefore be beneficial with an accurate, minimal invasive, method of transplantation to the pancreas parenchyma [19].

**Figure 1:**
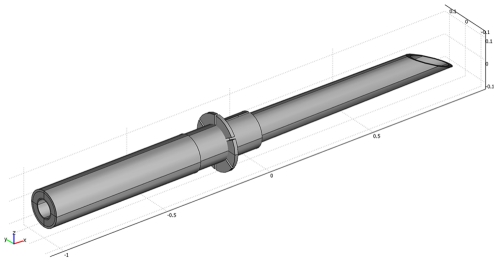
Principal design of the tip of the Extroducer. A principal drawing of the design of the distal tip is depicted with an intrusion depth limiting collar. Proximal to the intrusion depth limiting collar a electrolysis detachment zone is located. This image is previously published under the Creative Commons Attribution License in Lundberg et al, New endovascular method for transvascular exit of arteries and veins: developed in simulator, in rat and in rabbit with full clinical integration. *PLoS One* 5:e10449, 2010.

The Extroducer is designed for exiting the micro- or macro-vasculature throughout the body on both the arterial and the venous side (fig. 1) [20]. The design and concept of the device is associated with Seldingers original work describing the introducer [21]. A standard endovascular clinical catheter system, including an introducer, a guidecatheter and a microcatheter, is navigated within the vasculature to any target organ. Once the microcatheter is in the desired location within the microvasculature, the Extroducer system is advanced through the microcatheter. The Extroducer then safely penetrates the arterial or venous wall, as a “nanocatheter”, to reach the extravascular space, e.g. the parenchyma of any desired organ. The system thereby creates a working channel in order to be able to administrate or sample cells and substances to/from the extra-vasal space, and to make closure of the vessel wall safe even on the arterial side. To ensure the closure of the vessel wall, the most distal part of the Extroducer is detached and left behind. The rationale behind this technique is to combine minimal invasiveness of an endo-luminal approach with an accurate administration in a desired anatomical location.

The Extroducer is manufactured in nitinol; a nickel/titanium alloy with both memory and super elastic properties [22]. These special properties are the foundation to the use of nitinol in stent fabrication in clinical practice. Thus the excellent biocompatibility of nitinol [23] has been extensively studied, especially with respect to direct vascular inner wall interactions, i.e. endothelium [24] but no *in vivo* studies have been performed with nitinol penetrating the vascular wall. However, nitinol corrosive bi-products can have a negative effect on smooth muscle cells *in vitro* [25]. It should be noted that to achieve a cytotoxic effect, a continuous current was used over a nitinol wire for 24 hours thereby dissolving it into Titanium and Nickel ions.

### Study Design

The aim of this study was to evaluate long term effects of the trans-vessel wall technique where the distal portions of the Extroducer prototypes are detached in rabbits. Two primary endpoints were considered; stenosis at the site of intervention, ischemic complications distal to the intervention and late hemorrhagic complications. Further, a model of repeated endovascular intervention in the rabbit was established.

Since this is the first follow up study of the endovascular trans-vessel wall intervention the study was organized as a continuous enrollment of animals with successful interventions. A limitation was set to maximum two hours femoral introducer time to avoid ischemic damage in the hind limb of the rabbit. This time was chosen in accordance with previous experiences from endovascular treatment of children with similar size of artery and introducer sizes.

The interventions started with angiography to decide the most suitable places for interventions and then as many interventions as possible were performed in each subject within the time frame of two hours of femoral introducer time. New subjects where continuously enrolled to reach a total of 25 interventions for the study. The acute phase of intervention was studied in a previous article [20] and in the present study, we wanted to analyze the potential complications during the subacute and chronic phase. The follow up times where extended continuously from first five days, then 30 days and finally 80 days when follow ups and autopsies where performed without complications according to the primary endpoints.

We designed the study to assess complications following the trans-vessel wall technique in the vessel used for intervention and also distal to the intervention. Complications were assessed by follow-up angiography and histological examination as well as observation of the clinical status of the animal. Complications were considered to be hemorrhage at the interventional site at any time, stenosis at the interventional site, thrombosis at the interventional site and distal embolization. The secondary endpoint was efficacy of creating the working channel from within the vascular tree to the parenchyma of different organs.

## Results

In this study we show that it is possible to, by endovascular methods, reach several different parts of the body by perforating the vessel wall from the inside to out, thereby creating a working channel to the organ parenchyma without hemorrhagic complications during the acute- or chronic phase. The Extroducer and the trans-vessel wall technique are fully integrated with clinical standard material such as catheter systems and angiographical equipment. We show that neither stenosis formation, distal embolus formation, nor other adverse events occur with up to three months follow-up. The Extroducer interventions were performed in the superior mesenteric artery (SMA) (n = 9), the subclavian artery (SCA) (n = 14) and the external carotid artery tree (ECA) (n = 2).

We started with a total number of 25 primary trans-vessel wall interventions in 17 animals. Working channels were established in 100% of the cases without acute hemorrhagic or thrombo-embolic events, verified by DSA. Six of the interventions were aborted due to distal tip detachment failure with our hand-made relatively primitive electrolysis detachment method (leading to euthanasia of 3 animals and 3 that we were able to salvage to stay in the study). Two animals were euthanized by order of the veterinarian during the follow-up period, one because of a non-related pulmonary infection (but included and analyzed by histology) and the other since it fulfilled the behavioral abortion point criteria after 24 hours (excluded from the study). That animal was then autopsied and was found to have an embolic occlusion of the internal carotid artery without association to the Extroducer placement, but rather associated with the vascular navigation. The final distribution of followed up animals were as follows; two at the five days end point with only histological analysis of one, five at the 30 days end point and six at the 80 days end point with histological analysis of one (with pulmonary infection) resulting in a total number of 19 detached Extroducer tips (table 1).

**Table 1:** Distribution of analysed detached tips.

	SMA	SCA	ECA	No of animals examined at timepoint
5 days	1	2	0	2
30 days	4	3	0	5
80 days	3	5	2	6

The distal tip placement distribution in animals not excluded, plotted against time-points for follow-up. SMA = Superior Mesenteric Artery, SCA = Subclavian artery, ECA = External Carotid Artery.

**Figure 2:**
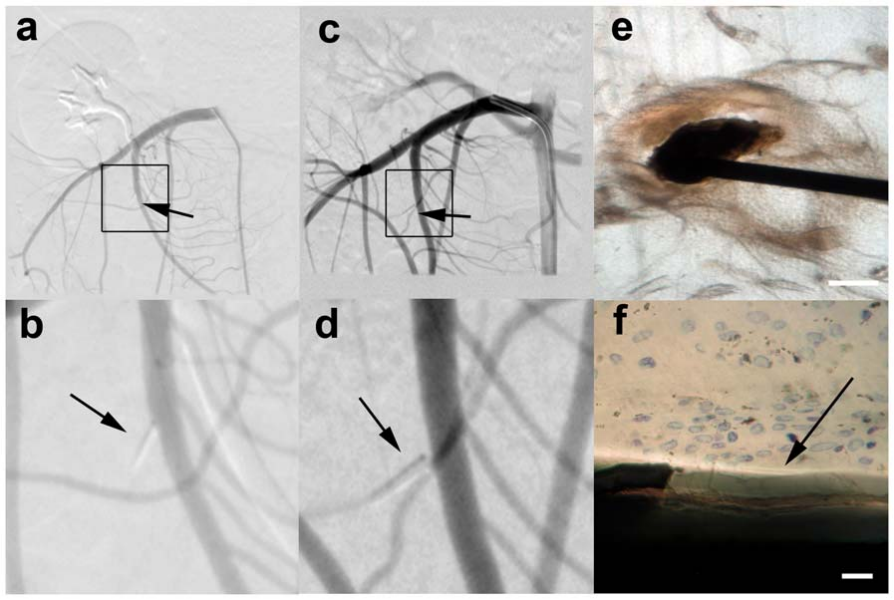
Follow Up of the Detached Distal Tips. In a. the initial follow up angiogram directly following detachment in the Superior Mesenteric Artery (SMA) is shown with a square marking the blow-up in b. Arrows indicate the detached distal tip. In c. an SMA angiogram, performed 80 days after the intervention in the same animal, is shown with a square indicating the blow-up in d. Arrows indicate the detached distal tip. In e. a microphotograph of a histological van Geeson and toulene blue staining prepared by grind-cutting with a detached tip *in situ*, is shown. Scale bar = 100 µm. The blow-up in f shows the parylene coating surrounding the detached tip which is marked by an arrow. Note that no fibrous response or inflammation is observable. Scale bar = 4 µm.

Behavioral monitoring of all animals were performed for signs of distress; especially from the prototypes placed in the SMA. Behavioural observations were performed five times per week and we observed no signs of distress in the animals followed during the observation period. We postulated that a possible thrombo-embolic complication in the SMA would have led to ischemia in the bowel which in turn would have given clear signs of distress for the animal and death. At the designated end time-points, cut down technique was used to place the introducer in the same femoral artery that had been used during the intervention. All follow-up introducer placements were successful. The follow-up DSAs were performed with at least two projection planes (fig. 2) and by 3D angiography. Philips proprietary software for the Allura XD20 was used to assess possible stenosis formation. We did not detect any stenosis or distal embolus at any follow-up time points. Further, we also performed navigations with microguide wires and microcatheters without complications distal to the detached distal tips.

In the follow up DSA, we also found that 4 of 19 (21%) of the detached tips were no longer placed through the vessel wall but had instead been “pushed” outward through the endothelium to the extra-vascular space immediately adjacent to the vessel penetration site. For prototype tips exhibiting this dislodgement through the endothelium, no vascular alterations or stenoses was observed.

Another interesting finding was that for the five prototypes detached precisely in the rotator plane of the humero-scapular joints in the SCA, tips were dislodged into the lumen of the vessel with the depth limiting collar pointed distally in the flow of blood. For all other tips, detached millimeters proximal or distal to the rotational plane through the SCA, this phenomena was not seen.

After the euthanasia procedures, we performed careful dissections to analyze macroscopic changes in the bowel, forelimb or facial muscles. All tissues examined where normal without signs of thrombo-embolic complications. To further analyze the effect of the detached prototypes, tissue samples were obtained *en bloc* with vessels and surrounding tissue.

**Figure 3:**
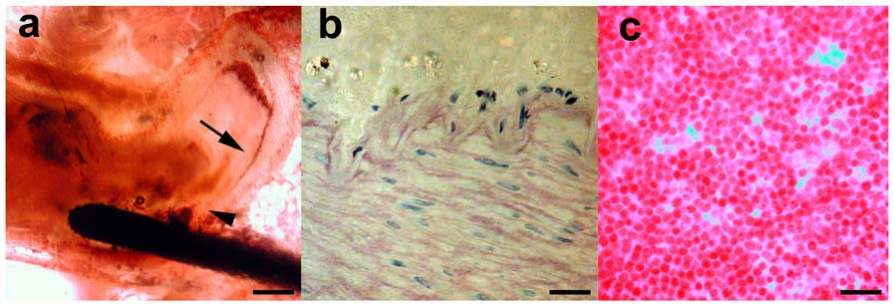
Histological Examination. After follow-up angiography, histological examinations were performed. In a. a microphotograph of a representative grind-cut section from the SMA stained with van Geeson and toulene blue, is shown (scale bar = 250 µm). In b. a high power field microphotograph of van Geeson and toulene blue with phase contrast stained endothelium adjacent to a deposit tip, is shown. No cellular alterations of the composition of the blood vessel is detected (scale bar = 30 µm). In c. a microphotograph of van Geeson stained lymph node from the mesentery adjacent to a detached tip, is shown. No signs of lymph node activation is seen (scale bar = 30 µm).

We performed histological analysis with the prototypes *in situ* by grind-cutting technique and then consulted an external, independent, evaluator with expertise in the field of titanium implants. The evaluation showed full biocompatibility with a very small fibrotic capsule (<1 µm) formed around the detached distal tips. No ongoing inflammation was observed around any of the detached tips. Around one (of the total number 14 left in place) implant three macrophages were indentified in the area of the detached distal tip. This was observed in a day five animal. The endothelia showed no signs of alterations adjacent to the detached tips. In conclusion, the biocompatibility of the trans-vessel wall detached nitinol tips were all comparable to titanium implants (fig. 3).

**Figure 4:**
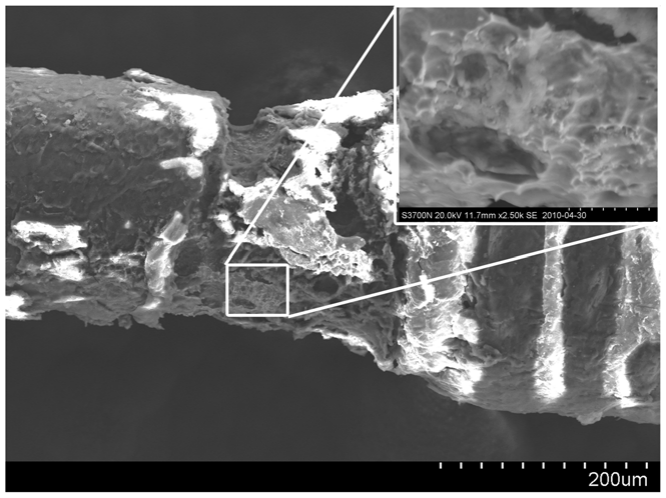
Scanning Electron Microscopy of Detachment Zone. For the devices that failed to detach, scanning electron microscopy was performed. In this figure an overview is shown of a failed detachment with a blow up from where spectroscopic analysis indicated formation of a passive layer, possibly by Titanium – Chloride ion formation.

Prototypes excluded due to failed detachment were analyzed by scanning electron microscopy (SEM) since monitoring of electric current gave limited prognostic information about failure to detach (fig. 4). In failed detachments, large amounts of chloride were observed by energy dispersive spectroscopy (EDS) of the surface (6–10 wt%) indicating formation of titanium chloride ions in a passive sheet, thus providing current transmission without electrolysis.

## Discussion

To realize the full potential of the trans-vessel wall technique, safety studies in several animals are an absolute necessity. In this study we show, for survival times up to 80 days in rabbit, that hemorrhage, stenosis formation or distal embolisation do not occur with this technique. Moreover, the biocompatibility of the trans-vessel wall implanted nitinol tips is excellent with no inflammatory activation in the surrounding tissues.

In previous studies, we show that, for cells capable of performing diapedesis, intra-arterial transplantation carries significant advantages with respect to engraftment compared to intravenous methods [13]. However, the selective intra-arterial method also has limitations in more specialized pathological conditions and/or specialized cell systems; *e.g.* insulin producing cells. Pre-clinical trials of specific intra-arterial transplantations with insulin producing cells have hitherto only showed negative results [15] and since open surgical options of accessing the pancreas parenchyma itself carries unacceptable risks [17] the portal vein approach has been developed [12]. In this study, the SMA-placed device could be considered a simulation of a retro-peritoneal intervention to reach the pancreas, thus this study provides safety data and rationale for a continuing development of the trans-vessel wall technique. Interventions aimed for the pancreas are not possible in rabbits and therefore such studies will be performed in swine. It should be noted that the usage of C-arm based computerized tomography reconstructions with for example Dyna-CT or XperCT technique in combination with 3D roadmapping would add substantial information about the relation of vessels and parenchyma, thereby facilitating directed parenchymal access with the trans-vessel wall technique. Another interesting new method is image fusion by previously acquired magnetic resonance imaging volumes with 3D roadmaps. All these new imaging techniques are transforming the field of endovascular intervention where the trans-vessel wall technique could potentially add a further tool in the toolbox of the interventional radiologists.

Since nitinol is in clinical use in a wide variety of applications with direct endothelial contact and in other types of implants, the bio-compatibility is well studied [22], [24]. Probably the best data set of compatibility comes from stents developed in nitinol which share at least the endothelial contact with the devices used in the present study. The present study is, however, to our knowledge the first study to describe the biocompatibility of nitinol when placed through the vessel wall layers and reaching the extravascular space. Our histological analysis shows minimal tissue reactions without signs of inflammation in the extra-cellular matrix or in the vessel walls. The design of the Extroducer prototypes evaluated in this study makes it possible to manufacture future devices also in bio-degradable material.

During the initial testing five prototypes, that were detached precisely in the rotator plane of the humero-scapular joints in the SCA, were dislodged into the lumen of the vessel. This phenomenon occurred due to the fact that the anterior extremities of the rabbit were flexed in a certain position to facilitate body fixation during the procedure and that the five Extroducer tips were detached exactly in the plane of movement of the joint. Subsequent analysis showed that the dislodgement of the penetrating tip occurred immediately after the procedure when the flexed anterior extremity regained its normal position. For the other prototypes detached within other segments of the SCA (millimeter more proximal or distal to the humero-scapular rotator plane) and in all other vascular segments tested in the body, this phenomenon was not observed. Thus, we do not consider it a problem since the target organs primarily are pancreas, CNS, heart and lung whereas the axillary cavity easily can be reached by percutaneous methods.

When analyzing the failed detachment tips by SEM we showed that a Titanium Chloride passive sheet was probably formed. Titanium chloride molecules can provide a surface area that lets electron pass through but without formation of soluble titanium or nickel ions. This problem probably occurred due to the relatively simple “home made” technique used for creating the insulation defect required for electrolysis detachment. There are, however, numerous commercially available solutions for detachment that can easily be integrated with the trans-vessel wall technique in industrialized manufacturing processes.

In conclusion this study shows that in large animals, the trans-vessel wall technique has a 100% success rate in creating an endovascular working channel to the parenchyma of any organ. Further, no stenosis, hemorrhage, embolus release or adverse tissue reactions are induced up to three months after the interventions. The benefit of the trans-vessel wall technique should be greatest in transplantations involving cells with limited capability of performing diapedesis, for example when transplanting insulin-producing cells in diabetes mellitus. The system can potentially also be used for local substance administration or tissue sampling, or for other diagnostic or therapeutic purposes in i.e. the CNS, pancreas, heart and lung.

## Materials and Methods

### Ethic statement

All animal studies were conducted according to Karolinska Institutet guidelines of animal experiments on small rodents and rabbits. The studies were approved by the Northern regional ethics committee for animal research in Stockholm, Sweden with reference number N08/09.

### Endovascular procedure

A total number of 17 New Zealand White rabbits were included in this study, divided into three time-points; 5 days, 30 days and 80 days. Surgical anesthesia was induced by subcutaneous injection of 0.5 ml/kg Hypnorm (fentanyl citrate 0.315 mg/ml, fluanisone 10 mg/ml, Janssen Pharmaceutical, Belgium) combined with 5 mg diazepam. An intravenous line was established followed by a bolus dose of Propofol and thereafter, the rabbit was intubated with a size 3 pediatric tube and connected to a Siemens 900 servo ventilator (Siemens Healthcare, London, United Kingdom). Continuous infusion of propofol and fentanyl was used to sustain surgical anesthesia.

All large animal angiography and endovascular intervention was performed with a Philips XD20 angiographical equipment (Philips medical system, the Netherlands). Visipaque 270 contrast agent (GE healthcare, USA), was used in all angiography applications.

The femoral artery was exposed surgically and a 4 French introducer was inserted in the artery (Terumo, USA). Under fluoroscopic and angiographic control, a 4 French Vertebral guiding catheter (Cordis Corporation, USA) was navigated to different parts of the vasculature of the rabbit. A Renegade micro-catheter (Boston Scientific, USA) was inserted within the guiding catheter and, together with a Transend Platinum Tip guidewire (Boston Scientific, USA), navigated under angiographic control to SMA, SCA and ECA branches. After having reached the desired target location, the guidewire was withdrawn from the microcatheter and the Extroducer, within a PTFE-190 Sub-Lite wall tubing (Agnthos AB, Stockholm, Sweden), was inserted and deployed through the arterial wall and detached with electrolysis.

Maximal femoral artery introducer time was two hours. After the interventions, gentle pressure was applied on the femoral artery for 15 minutes, followed by Coseal™ application and another 15 minutes of pressure. The surgical incision was then sutured and propofol infusion was discontinued. On reestablishment of the coughing reflex, extubation was performed and the animals were allowed to recover in their home cages. Behavioral monitoring was performed five times per week.

When performing follow-up angiography, an introducer was placed in the femoral artery under general anesthesia (see above) and a diagnostic angiography was performed using a 4 French diagnostic catheter. Thereafter the animal was sacrificed by a lethal dose of Penthotal and the vasculature and organs, where interventions had been performed, were dissected under surgical microscope and samples for histological analysis were taken.

### Histological examination

We performed histochemical analysis of blocks of tissue containing the detached tips. The appropriate tissue was dissected with fluoroscopic guidance. The tissue blocks were then mounted in plastic and cut by specialized ground cutting technique providing the possibility of leaving the metal tip *in situ*. Staining was performed with van Geeson and toulene blue on grind cut blocks and then visualized by light-microscopy.

An external, independent, evaluator from the department of pathology, Karolinska University Hospital, Sweden, with expertise on biological implants, was consulted for assessment of the histological results. Samples were blinded with respect to time and location for the evaluator and the benchmark for biocompatibility were set as the tissue reaction produced by titanium implants and counting of macrophages around the implant.

### Scanning Electron Microscopy

For SEM investigations a Hitachi S-3700N was used (Hitachi AB, Sweden). The SEM was connected to a Bruker AXS XFlash Detector 4010 for spectroscopical analysis (Bruker Corp. US). Samples were cleaned prior to analysis by rinsing with tap water.
